# Left parathyroid carcinoma with secondary hyperparathyroidism: a case report

**DOI:** 10.1186/s12902-023-01370-x

**Published:** 2023-05-16

**Authors:** Ko Yokoyama, Nobuyasu Suganuma, Yasushi Rino

**Affiliations:** Department of Surgery, Yokohama City University, Yokohama, Japan

**Keywords:** Parathyroid carcinoma, Hyperplasia, Hypercalcemia, Hyperparathyroidism, Parathyroid hormone

## Abstract

**Background:**

Parathyroid carcinoma is a rare disease with a frequency of 0.005% of all malignancies [1, 2]. Various aspects of its pathogenesis, diagnosis, and treatment remain poorly understood. Furthermore, cases with secondary hyperparathyroidism are fewer. In this case report, we describe a case of left parathyroid carcinoma with secondary hyperparathyroidism.

**Case presentation:**

The patient was a 54-year-old woman who had been on hemodialysis since the age of 40 years. At 53 years of age, her calcium levels were high, and she was diagnosed with drug-resistant secondary hyperparathyroidism and was referred to our hospital for surgical treatment. Blood tests revealed calcium levels of 11.4 mg/dL and intact parathyroid hormone (PTH) levels of 1007 pg/mL. Neck ultrasonography revealed a 22-mm large round hypoechoic mass, partially indistinct margins, and D/W ratio > 1 at the left thyroid lobe. Computed tomography scans revealed a 20-mm nodule at the left thyroid lobe. No enlarged lymph nodes or distant metastases were noted. ^99m^Tc-hexakis-2-methoxyisobutylisonitrile scintigraphy revealed an accumulation at the superior pole of the left thyroid lobe. Laryngeal endoscopy revealed paralysis of the left vocal cord, signifying recurrent nerve palsy due to parathyroid carcinoma. Based on these results, a diagnosis of secondary hyperparathyroidism and suspected left parathyroid carcinoma was made, and the patient underwent surgery. Pathology results revealed hyperplasia in the right upper and lower parathyroid glands. The left upper parathyroid gland showed capsular and venous invasion, and the diagnosis was left parathyroid carcinoma. At 4 months post-surgery, calcium levels improved to 8.7 mg/dL and intact PTH levels to 20 pg/mL, with no signs of recurrence.

**Conclusions:**

We report a case of left parathyroid carcinoma associated with secondary hyperparathyroidism. Concomitant secondary hyperparathyroidism may cause mild hypercalcemia compared to parathyroid carcinoma alone due to the added modification of dialysis. Although our patient also presented with mild hypercalcemia, a D/W ratio > 1 on preoperative echocardiography and presence of recurrent nerve palsy on laryngoscopy led to the suspicion and treatment of parathyroid carcinoma preoperatively.

## Background

Parathyroid carcinoma is a rare disease with a frequency of 0.005% of all malignancies [1, 2]. Various aspects of its pathogenesis, diagnosis, and treatment remain poorly understood. Parathyroid carcinoma typically presents as a nodule and should therefore be differentiated from parathyroid adenoma, parathyroid or thyroglossal cyst, multinodular goiter, thyroiditis, thyroid adenoma and carcinomas [3]. Although puncture aspiration cytology or needle biopsy of a tumor is an important diagnostic test, if the tumor is parathyroid carcinoma, those tests are contraindicated, as they cause spread of cancer cells when the capsule is damaged. Hypercalcemia is an important finding in the diagnosis of parathyroid carcinoma, serum calcium levels are > 12 mg/dL or more than 3-4 mg/dL above the upper limit of normal range. However, very rare cases of secondary parathyroid function should be noted, as they are modified by dialysis. In this case report, we describe a case of left parathyroid carcinoma with secondary hyperparathyroidism.

## Case presentation

### Patient characteristics

The case was a 54-year-old woman, referred to our department for hypercalcemia. Her past medical history included chronic renal failure (maintenance dialysis), hypertension, and hyperuricemia. No significant family history was noted. The patient had been on hemodialysis since the age of 40 years. In April 2020 (at the age of 53 years), poor control of blood calcium levels was observed, and high-dose maxacalcitol was commenced in October 2020, but no improvement was observed. In December 2020, she was referred to her previous doctor with a diagnosis of drug-resistant secondary hyperparathyroidism and was referred to our hospital for close examination and treatment.

**Fig. 1:**
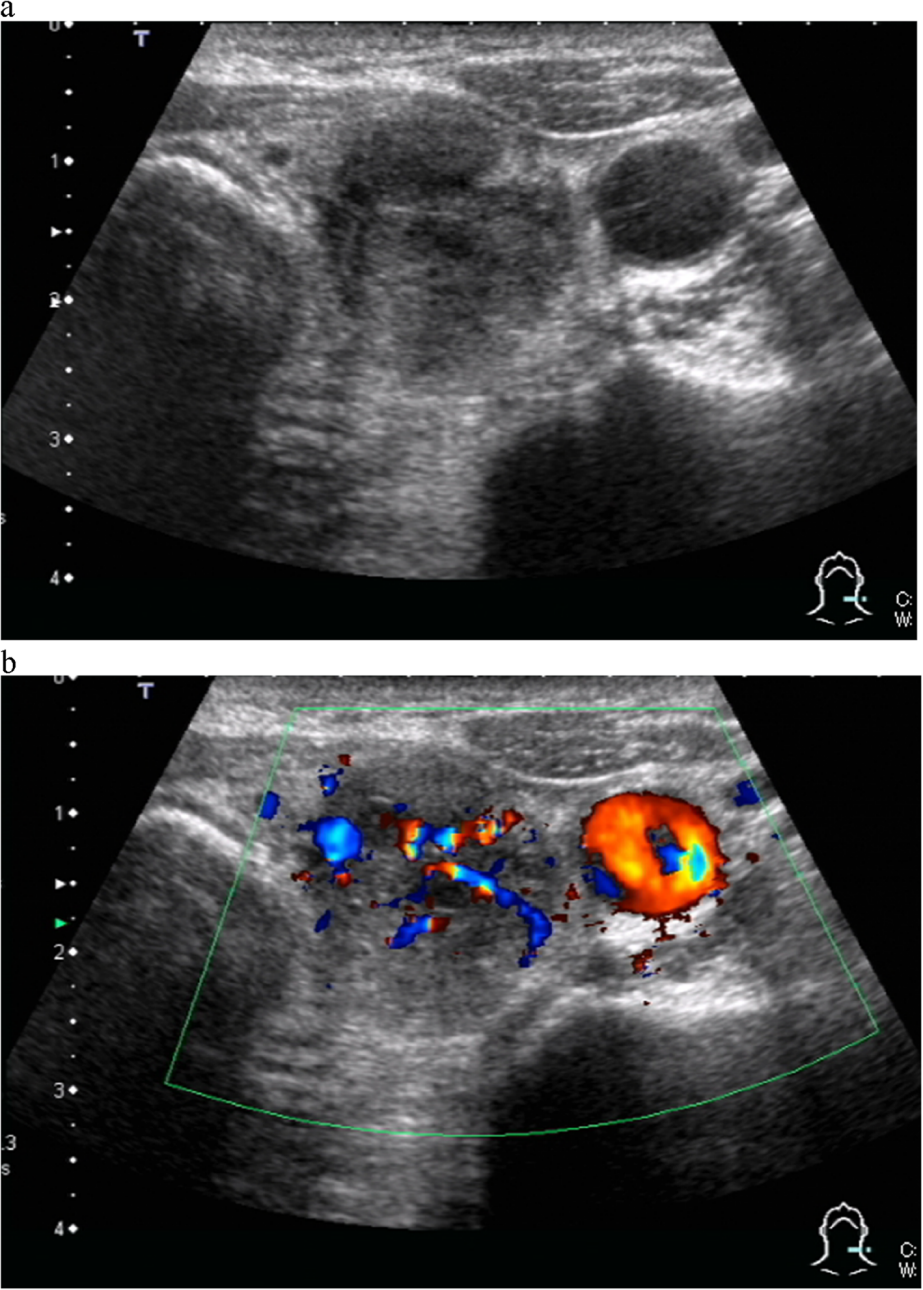
Neck ultrasonography. **a** Hypoechoic mass measuring 21 mm in size with irregular and partly indistinct margins, D/W ratio > 1. **b** Admission of abundant blood flow

On admission, the patient had a height of 155.0 cm, weight of 56.7 kg, body mass index of 23.6 kg/m^2^, and no palpable mass in the neck. Her blood test results are presented in Table 1. High creatinine (8.73 mg/dL), calcium (11.4 mg/dL), phosphorus (9.2 mg/dL), and intact parathyroid hormone (PTH) levels (1007 pg/mL) were observed. Neck ultrasonography revealed a hypoechoic mass measuring 30.3 × 23.7x21.8 mm in size with irregular and partly indistinct margins, D(23.7)/W(21.8) ratio > 1, and abundant blood flow at the dorsal surface of the left thyroid lobe, outside the capsule (Fig. 1). A 10-mm large well-defined hypoechoic mass was observed at the dorsal surface of the right thyroid lobe, outside the capsule.

**Table 1:** Blood test results

Blood test	Result	Reference value	Unit
White blood cell	4400	3500–9500	/mm^3^
Hemoglobin	13.2	13.0–18.0	g/dL
Platelet count	11.9	15.0–35.0	10^4^/μL
Total protein	6.4	6.0–8.0	g/dL
Albumin	3.5	3.9–4.9	g/dL
Urea nitrogen	77	6.0–20.0	mg/dL
Creatinine	8.73	0.4–1.3	mg/dL
Sodium	144	135–150	mmoL/L
Potassium	5.5	3.5–5.0	mmoL/L
Chloride	106	103–113	mmoL/L
Calcium	11.4	8.7–11.0	mg/dL
Inorganic phosphate	9.2	1.9–4.7	mg/dL
C-reactive protein	0.07	0.0–0.5	mg/dL
Asparate aminotransferase	9	6–40	U/L
Alanine aminotransferase	11	6–37	U/L
Alkaline phosphatase	85	96–284	U/L
Lactate dehydrogenase	27.7	105–210	U/L
Total bilirubin	0.4	0.2–1.2	mg/dL
Thyroid-stimulating hormone	2.160	0.35–4.94	μIU/m
Free Triiodo thyronine	2.33	1.68–3.67	pg/mL
Free Thyroxine	0.92	0.7–1.48	ng/dL
Thyroglobulin	13.90	< 33.7	ng/mL
Thyroglobulin antibody	11.3	< 4.11	IU/mL
Intact parathyroid hormone	1007	10–65	pg/mL

### Imaging findings

**Fig. 2:**
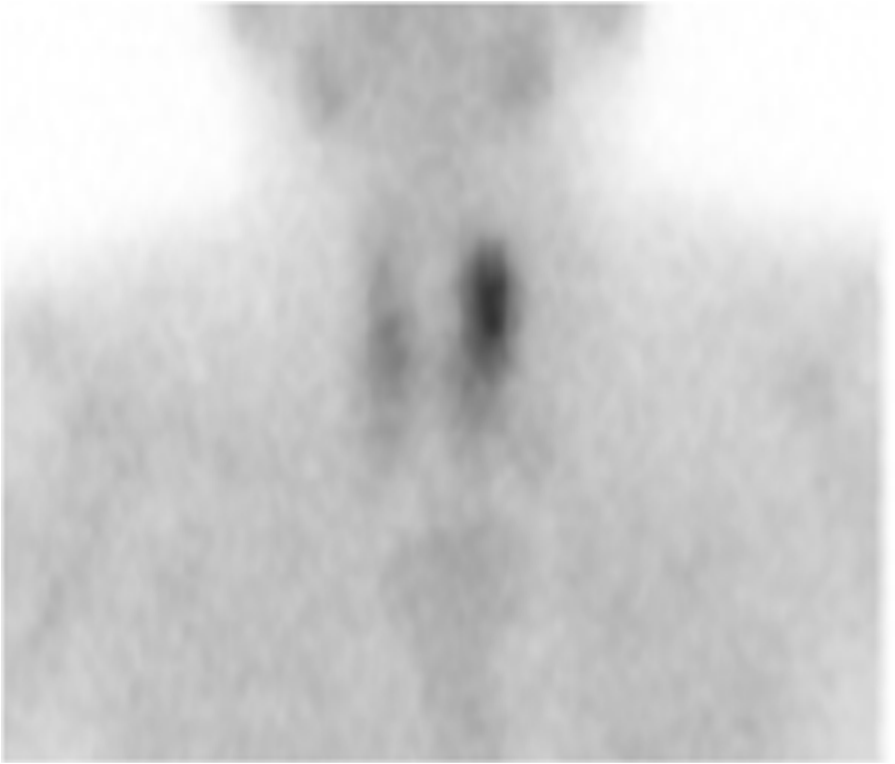
^99m^Tc-hexakis-2-methoxyisobutylisonitrile scintigraphy (Early phase))

Cervical computer tomography (CT) scan revealed a 20-mm nodule at the dorsal surface of the left thyroid lobe and a 10-mm enlarged parathyroid gland at the dorsal surface of the right thyroid lobe. ^99m^Tc-hexakis-2-methoxyisobutylisonitrile scintigraphy revealed an accumulation at the upper pole of the left thyroid gland (Fig. 2). Laryngeal endoscopy revealed paralysis of the left vocal cord (median fixation).

### Treatment plan

**Fig. 3:**
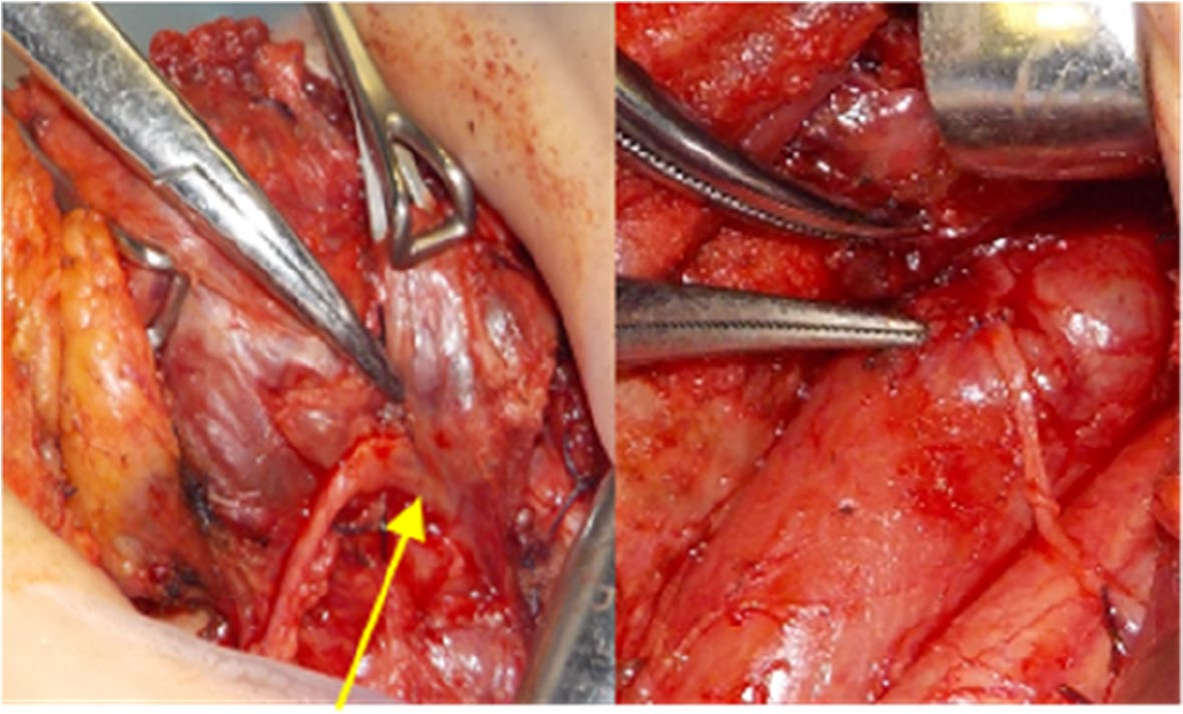
Surgical findings. Lt: Tumor invading the left recurrent nerve. Rt: Excision of the left thyroid lobe including the recurrent nerve and reconnection with the ansa cervicalis

Based on the test results, we suspected secondary hyperparathyroidism with left parathyroid carcinoma, leading us to perform total parathyroidectomy, peritracheal lymph node dissection, combined thyroidectomy, left recurrent nerve resection and reconstruction (left recurrent nerve – ansa cervicalis), and autograft of the parathyroid to right upper arm in February 2021 (Fig. 3).

### Surgical findings

The left upper parathyroid tumor was located 10 mm centrally from the left recurrent nerve and invading inwards circumferentially. We thus excised the left thyroid lobe, including the recurrent nerve, and reconnected it with the ansa cervicalis with two stitches of 6–0 Proline(Johnson & Johnson, City of New Brunswick, USA) and Beriplast(CSL Behring, King of Prussia, USA).

### Postoperative course

The anterior cervical drain was removed at postoperative day (POD) 2, and the patient was discharged at POD 3 without complications.

### Pathology results

**Fig. 4:**
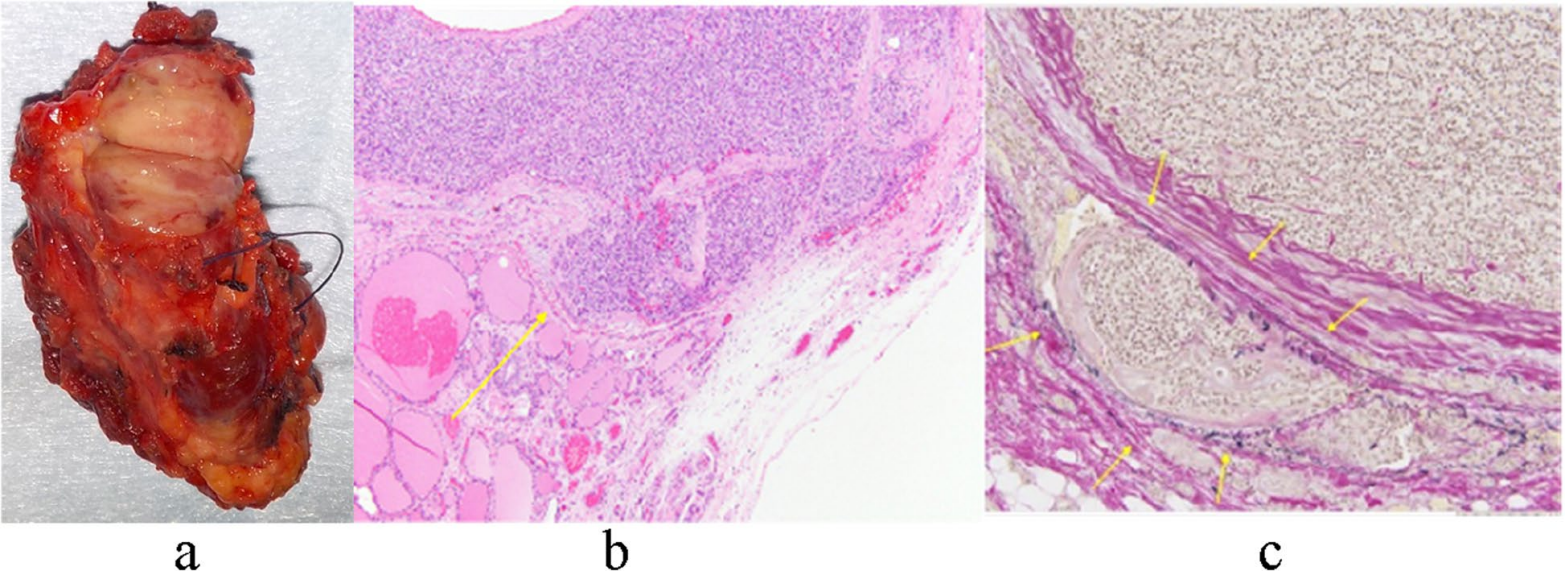
Pathological specimens. **a** (macro): Left thyroid lobe including a milky white substantial mass measuring 18 × 13 × 18 mm in the upper pole. **b** (hematoxylin & eosin [HE] stain): Large round nucleus, fenestrated-to-dense growth of principal cells with pale acidophilic cytoplasm, and a fibrous capsule covering the mass. Acidophilic cells with acidophilic cytoplasm proliferating in a fenestrated-to-dense manner are observed. **c** (Elastin van Gieson [EVG] stain): EVG stain showed some venous invasion

Macroscopic pathology analysis revealed a left thyroid lobe measuring 59 × 30 × 20 mm, including a milky white substantial mass measuring 18 × 13 × 18 mm in the upper pole. The left lower parathyroid gland was normal (Fig. 4a). We were unable to measure the left parathyroid due to cancer invasion. The right upper and lower parathyroid glands were 15 × 12 × 7 mm and 7 × 7 × 7 mm in size and weighed 450 mg and 90 mg, respectively.

Microscopic pathology analysis revealed that the left upper mass consisted of a large round nucleus, fenestrated to dense growth of principal cells with pale acidophilic cytoplasm, and a fibrous capsule covering the mass. We observed acidophilic cells with acidophilic cytoplasm proliferating in a fenestrated to dense manner (Fig. 4b). Some parts of the lesion appeared to proliferate beyond the fibrous capsule. Elastin van Gieson staining revealed venous infiltration in some areas, suggesting vascular invasion (Fig. 4c); therefore, the diagnosis was parathyroid carcinoma. No lymph node metastasis was observed(0/1). The other parathyroid glands exhibited nodular proliferation of principal cells and eosinophilic cells forming a fenestrated to follicular structure. No obvious normal rim was noted at the specimen margins. The diagnosis was hyperplasia of the right upper and lower parathyroid gland.

### Postoperative outpatient course

At 1 month postoperatively, calcium and iPTH levels were 9.5 mg/dL and 15 pg/mL, respectively, with no subsequent increase. At 25 months postsurgery, no apparent evidence of recurrence was observed. Table 2 shows pre and postoperative calcium and iPTH levels.

**Table 2:** Pre and postoperative calcium and Intact PTH levels

Date	12/2020 (Preoperative)	2/2021 (POD1)	3/2021 (POM1)	6/2021 (POM4)	12/2021 (POM10)	9/2022 (POM19)	3/2023 (POM25)
Calcium (mg/dL)	11.4 (mg/dL)	8.9 (mg/dL)	9.5 (mg/dL)	8.7 (mg/dL)	8.7 (mg/dL)	9.5 (mg/dL)	9.0 (mg/dL)
Intact PTH (pg/mL)	1007 (pg/mL)	10 (pg/mL)	15 (pg/mL)	20 (pg/mL)	23 (pg/mL)	25 (pg/mL)	20 (pg/mL)

*PTH* Parathyroid hormone, *POD* Post operative day, *POM* Post operative month

## Discussion and conclusions

Parathyroid carcinoma is a rare disease, accounting for only 0.005% of all cancers and 0.3–5.6% of primary hyperparathyroidism. Various aspects of its pathogenesis, diagnosis, and treatment remain poorly understood [1, 2]. Moreover, puncture aspiration cytology and needle biopsy are contraindicated, as they cause spread of cancer cells when the capsule is damaged, rendering it difficult to make a definitive diagnosis before surgery. Characteristic clinical findings include palpation of neck mass (sensitivity of 50% and specificity of 100%), generalized fibrous osteitis (sensitivity of 50% and specificity of 38%), and serum calcium levels > 12 mg/dL (sensitivity of 83% and specificity of 69%) [4–9]. Other findings suggestive of parathyroid carcinoma include the D/W ratio on neck ultrasonography and invasion of the thyroid gland and recurrent nerve [8]. In the case reported herein, none of the aforementioned clinical features was present. Although the D/W ratio was > 1, and recurrent nerve palsy was observed, we were able to remove the tumor without exposing it by suspecting parathyroid carcinoma preoperatively.

With regard to pathological diagnosis, parathyroid carcinoma is often grayish-white, hard, and has a gross irregular shape. The four histological diagnosis criteria proposed by Schantz and Castleman are (1) formation of a thick fibrous capsule within the tumor, (2) fenestrated arrangement of tumor cells, (3) nuclear fission image, and (4) capsular or vascular invasion [10]. However, as the first three features are also observed in adenomas, it is difficult to differentiate parathyroid adenomas from carcinomas based solely on histopathological findings [11]. To distinguish parathyroid adenomas and carcinomas, blood test findings may be useful. In parathyroid carcinoma, serum calcium levels are > 12 mg/dL or more than 3–4 mg/dL above the upper limit of normal range. In contrast, in parathyroid adenoma, calcium levels are only 1 mg/dL above the upper limit. Serum PTH levels also differ between parathyroid carcinomas and parathyroid adenomas. In parathyroid carcinoma, serum PTH levels are 3–10 times the upper limit of normal range, whereas in parathyroid adenoma, serum PTH levels are rarely more than twice the upper limit of normal range [12]. In this case, the serum calcium level was not above 12 mg/dl, partly due to hemodialysis.

Only 37 cases of parathyroid carcinoma associated with secondary hyperparathyroidism have been reported in the English literature since Berland et al.’s report in 1982 [13]. The 37 cases are summarized in Table 3 [13–46]. The mean age of the patients was 50.5 years. Of these cases, 18 were men and 18 were women (one has no gender indicated in the text), indicating approximately equal sex distribution. Regarding the three clinical features listed above, only one case (no other description) presented with a palpable neck mass, 17 cases (65.4%) exhibited symptoms suggestive of fibrotic osteitis, and 25 cases (80.6%) presented with serum calcium levels > 12 mg/dL. PTH levels were high in 29 patients (100%), although measurement methods differed in some cases. In patients with parathyroid carcinoma associated with secondary hyperparathyroidism undergoing dialysis, the characteristic clinical findings observed only in parathyroid carcinoma do not necessarily apply due to the additional modifications caused by dialysis.

**Table 3:** Cases of parathyroid carcinoma associated with secondary hyperparathyroidism

Case No	Author	Age	Sex	Fibrous osteitis	Calcium	PTH	Peripatetic infiltration	Recurrence	Outcomes
1	Berland et al. [13]	62	F	Yes	9.2	1820		None	Good
2	Anderson et al. [14]	44	F	None	High		None		Bad
3	Ireland et al. [15]	34	M	Yes	12.3	1043	Lung		Bad
4	Sherlock et al. [16]	42	F	None	12.9	10 (PTH-C)	None	None	Good
5	Krishna et al. [17]	64	F	Yes	High	High	None		Good
6	Kodama et al. [18]	53	F	Yes	11.1	121.4	None	None	Good
7	Iwamoto et al. [19]	46	M	Yes	9.6	24.2 (PTH-C)	Laryngeal nerve branch		
8	Iwamoto et al. [19]	55	F	Yes	9.4	98.3 (PTH-C)	Yes		
9	Rademaker et al. [20]	46	F						Good
10	Rademaker et al. [20]	52	F						Good
11	Tominaga et al. [21]	46	F	Yes	6.1	956	Thyroid gland, cervical lymph node, pulmonary metastasis		
12	Miki et al. [22]	40	F		7.8	High			Good
13	Liou et al. [23]	64	M		14.7	High			Good
14	Tseng et al., [24]	20	F	Yes	12.7	1143	Lung	Pulmonary metastasis	Bad
15	Takami et al. [25]	55	F		10.9	High			Good
16	Jayawardene et al. [26]	75	F		High	High			Good
17	Kuji et al. [27]	51	M						
18	Zivaljevic et al. [28]	69	M	Yes	High	High			Good
19	Srouji et al. [29]	27	F	Yes	11.2	1405			Good
20	Khan et al. [30]	33	M		10.6		Bone, lung		Good
21	Bossola et al. [31]	52	F	None	12.4	1366	None	None	Good
22	Babar-Craig et al. [32]	55	M						
23	Falvo et al. [33]	61	M						Good
24	Tkaczyk et al. [34]	55	M						
25	Diaconescu et al. [35]	48	M	Yes	10.4	710	None		Good
26	Nasrallah et al. [36]	53	M	None	11.1	324	Laryngeal nerve branch	None	Good
27	Kim et al. [37]	57	M	Yes	10.6	1278	None	None	Good
28	Pappa et al. [38]	45	M	None	10.6	1422	None	None (postoperative radiation)	Good
29	Curto et al. [39]	59	F	None	14	1544	Lung	None	Good
30	Shen et al. [40]	70	M	None	15.11	197	None	None	Good
31	Won et al. [41]	46		None	9.8	1399	None	None (postoperative radiation)	Good
32	Cappellacci et al. [42]	51	M	None	10.7	2000	None	None	Good
33	Malipedda et al. [43]	53	M	Yes	12.5	3360	None	None	Good
34	Kada et al. [44]	48	F	Yes	8.9	830	Esophageal mucosa muscle plate	None	Good
35	Chen et al. [45]	49	M	Yes	High	1483	None	None	Good
36	Radu et al. [46]	35	M	Yes	11.6	804	None	None	Good
37	Radu et al. [46]	55	F	Yes	13.2	1283	None	None	Bad

*PTH* Parathyroid hormone

Table 4 presents a comparison of the age, sex ratio, serum calcium level, PTH level, and presence of bone lesions in patients with and without secondary hyperparathyroidism [47–49]. No obvious differences were noted in age, sex ratio, PTH level, or presence of bone lesions between groups. Serum calcium levels were lower in patients with than in those without secondary hyperparathyroidism, presumably due to the removal of calcium by hemodialysis. In addition, hypercalcemia is also observed in 20–30% of patients with malignancies other than parathyroid carcinoma, caused by the release of PTHrP(parathyroid hormone-related peptide) [50]. For these reasons, imaging tests are crucial when parathyroid carcinoma is suspected. In many cases, CT scans were performed for uncontrolled hypercalcemia, and large nodules were observed around the thyroid gland, leading to surgical resection. However, it is difficult to distinguish between an adenoma and cancer based on the size of the parathyroid gland on CT. In several cases, a malignant tumor was observed in the postoperative pathology results after a preoperative diagnosis of secondary hyperparathyroidism only. If parathyroid carcinoma is not suspected preoperatively, it may be disseminated as described above. Therefore, we were able to resect the tumors while considering the risk of dissemination.

**Table 4:** Comparison of secondary hyperparathyroidism and parathyroid cancer (37 cases) with parathyroid cancer only (203 cases)

	Secondary hyperparathyroidism + parathyroid carcinoma (37 cases)	Parathyroid carcinoma (203 cases) [47]
Age	50.5	49.17
Sex ratio	18 male: 18 female	93 male: 110 female
Serum calcium level (mg/dL)	11.13 (mg/dL)	15.23 (mg/dL)
PTH (pg/mL)	1224 (pg/mL)	1369 (pg/mL)
Bone lesions (%)	65.4% (17 of 26 cases)	Under 70% [48, 49]

*PTH* Parathyroid hormone

In conclusion, we report a very rare case of left parathyroid carcinoma associated with secondary hyperparathyroidism. D/W ratio > 1 on preoperative echocardiography and presence of recurrent nerve palsy on laryngoscopy led to the suspicion and treatment of parathyroid carcinoma preoperatively.

## Data Availability

The datasets used and analyzed during the current study are available from the corresponding author on reasonable request.

## Abbreviations

CT: Computed tomography
POD: Postoperative day
PTH: Parathyroid hormone
